# Supplementary material for: A novel approach to co-expression network analysis identifies modules and genes relevant for moulting and development in the Atlantic salmon louse (Lepeophtheirus salmonis)
Source: BMC Genomics. 2021 Nov 18;22:832. doi: 10.1186/s12864-021-08054-7 (PMC8600823; doi:10.1186/s12864-021-08054-7)
Supplement: Supplementary file 1 — Additional file 1 The methods used to estimate the power parameter and calculate the module preservation statistics. [file 12864_2021_8054_MOESM1_ESM.pdf]

Supplementary methods and information for gene co-expression network construction

Zhaoran Zhou

Correspondence:
zhaoran.zhou@uib.no
Department of Informatics & Sea Lice Research Centre, University of Bergen, Thormøhlensgate 55, Bergen, Norway
Full list of author information is available at the end of the article

Estimation of power parameter of the scale-free network transformation

In this study, we choose the power parameter β from integers ranging from 1 to 20, using the criterion recommended by [1],i.e. we choose the smallest β that results in approximate scale-free topology as measured by the scale-free topology fitting index R^2. The scale-free topology fitting index is defined based on the frequency distribution of the connectivities in the network. The frequency distribution of network connectivity is calculated based on discretizing connectivities, and the discretized connectivity (dk) is defined as:

$$dk = discretize(k, no.bins), \tag{1}$$

where k represents the vector of network connectivities (see the formula-3 and formula-4 in the paper), and the function $discretize()$implements a equal-width discretization method to transform a numeric vector (k) to a vector (dk) of equal length whose components indicate the bin number into which the value falls. $no.bins$ represents the number of equal-width bins, and it can be set depending on the specific situations or it can be defined as:

$$no.bins = as.integer(\sqrt{m}), \tag{2}$$

where m is the number of observations, i.e. the number of components in the vector k. The frequency distribution of connectivity (p.Connectivity) can be defined as:

$$p.Connectivity = p(dk) = p(discretize(k, no.bins)). \tag{3}$$

A network exhibits scale-free topology if its frequency distribution $p(dk)$ follows a power law:

$$p(dk) = N * k^{-\gamma}, \tag{4}$$

where N and γ denote positive real numbers. The logarithm of forumla (4) is:

$$log(p(dk)) = -\gamma * log(k) + log(N), \tag{5}$$

indicating that there exists a straight line relationship between $log(p(dk))$ and $log(k)$ in a scale-free network, and the slope of the line should be negative. Therefore, the scale-free topology fitting index (R^2) can be defined to measure the extent of a straight line relationship between $log(p(dk))$ and $log(k)$:

$$ScaleFreeFit(no.bins) = R^2 = cor(log(p(dk)), log(BinNo)), \tag{6}$$

where $BinNo = (1, 2, ..., no.bins)$. Networks with a scale-free topology fitting index R^2 close to 1 are thus can be defined to be approximately scale free. Since the slope of the regression line between $log(p(dk))$ and $log(k)$ is negative, we multiplied R^2 by -1, i.e. we checked the signed R^2.

According to [1], there is a natural trade-off between maximizing scale-free topology fitting index (R^2) and maintaining a high mean number of connectivities. Therefore, it is recommended that only those power parameter values leading to a network satisfying scale-free topology at least approximately ($signedR^2 > 0.80$) should be considered. And the average of network connectivities should be high, i.e, choose the lowest power parameter β that results in approximate scale-free topology.

Statistics for module preservation analysis

The package provided five module connectivity-based preservation statistics to compare the module connectivity patterns changes between the reference network and test network: $cor.kIM$, $cor.kME$, $cor.kMEall$, $cor.cor$ and $cor.MAR$. For a given module, $cor.kIM$ is the correlation of intramodular connectivities of nodes in the module between the reference network and test network:

$$cor.kIM^{(q)} = cor(kIM^{[ref](q)}, kIM^{[test](q)}), \tag{7}$$

where the superscript $[ref](q)$ denotes the module q in the reference network, and $[test](q)$ denotes the module q in the test network, i.e $kIM^{[ref](q)}$ denotes the intramodular connectivities restricted to the nodes in module q in the reference network.

$cor.kME$ specifies the correlation of module memberships of nodes in the module between the reference network and test network, while $cor.kMEall$ calculates the analogous correlations for all nodes between the two networks:

$$cor.kME^{(q)} = cor_{i\in M_q}(kME_i^{[ref](q)}, kME_i^{[test](q)}) \tag{8}$$

$$cor.kMEall_i^{(q)} = cor(kME_i^{[ref](q)}, kME_i^{[test](q)}). \tag{9}$$

$cor.cor$ measures the correlation of the maximum adjacency ratio (MAR) of nodes in the module between the reference network and test network:

$$cor.cor^{(q)} = cor(vectorizeMatrix(r^{[ref](q)}), vectorizeMatrix(r^{[test](q)})). \tag{10}$$

$cor.MAR$ measures the correlation of the maximum adjacency ratio (MAR) of nodes in the module between the reference network and test network:

$$cor.MAR^{(q)} = cor(MAR^{[ref](q)}, MAR^{[test](q)}). \tag{11}$$

The maximum adjacency ratio (MAR) of node i is defined as follow:

$$MAR_i = \frac{\sum_{j\neq i}(A_{ij})^2}{\sum_{j\neq i}(A_{ij})} = \frac{\sum_{j\neq i}(A_{ij})^2}{k_i}, \tag{12}$$

where $k_i = \sum_{j\neq i} A_{ij}$.

There are four density-based preservation statistics for measuring preservation of module density:$meanCor$, $meanAdj$, $propVarExpl$ and $meanKME$. For a given module, $meanCor$ represents the mean correlation in the test network multiplied by the sign of the corresponding correlations in the reference network:

$$meanCor^{[test](q)} = mean\left(vectorizeMatrix(sign(r_{ij}^{[ref](q)})r_{ij}^{[tset](q)})\right), \tag{13}$$

where $r_{ij}^{[ref](q)}$ denotes the correlation matrix restricted to the nodes in module q in the reference network.

$meanAdj$ measures the module density in the test network:

$$meanAdj^{[test](q)} = Density^{[test](q)} = mean\left(vectorizeMatrix(A^{[test](q)})\right). \quad (14)$$

$propVarExpl$ is defined as proportion of variance explained by the module eigenvector, and can be calculated by as mean squared of kME :

$$propVarExpr^{[test](q)} = mean_{i \in M_q}\left((kME_i^{[test](q)})^2\right) \quad (15)$$

$meanKME$ is defined as sign-aware module membership:

$$meanKME^{[test](q)} = mean_{iinM_q}\left(sign(kME_i^{[test](q)})kME_i^{[test](q)}\right) \quad (16)$$

Furthermore, module preservation statistics based on permutation tests can be calculated in WGCNA to assess the significance of observed module preservation statistics. The module labels in the test network are randomly permuted to calculate the corresponding preservation statistics. For a statistic labeled by index a, the corresponding Z_a statistic is defined:

$$Z_a = \frac{obs_a - \mu_a}{\sigma_a}, \quad (17)$$

where obs_a represents the observed value of the statistic a, and μ_a and σ_a is the mean and standard deviation value obtained from the permutation respectively.

Finally, the connectivity-based preservation Z statistics and the density-based preservation Z statistics are aggregated into summary preservation statistics $Z_{connectivity}$ and $Z_{density}$:

$$Z_{connectivity} = median(Z_{cor.kIM}, Z_{cor.MAR}, Z_{cor.kME}, Z_{cor.kMEall}, Z_{cor.cor}) \quad (18)$$

$$Z_{density} = median(Z_{meanCor}, Z_{meanAdj}, Z_{propVarExpl}, Z_{meanKME}) \quad (19)$$

Since we are interested in the preservation of both connectivity pattern and density pattern of a module, the summary preservation statistics were assigned equal

weight in the composite Z summary statistic:

$$Z_{summary} = \frac{Z_{connectivity} + Z_{density}}{2}. \tag{20}$$

More details can be found in [2].

References

1. Zhang, B., Horvath, S.: A General Framework for Weighted Gene Co-Expression Network Analysis. Statistical Applications in Genetics and Molecular Biology **4**(1) (2005). doi:10.2202/1544-6115.1128
2. Horvath, S.: Evaluating Whether a Module is Preserved in Another Network. In: Weighted Network Analysis Applications in Genomics and Systems Biology, pp. 207–245 (2011)
