# Supplementary material for: A novel approach to co-expression network analysis identifies modules and genes relevant for moulting and development in the Atlantic salmon louse (Lepeophtheirus salmonis)
Source: BMC Genomics. 2021 Nov 18;22:832. doi: 10.1186/s12864-021-08054-7 (PMC8600823; doi:10.1186/s12864-021-08054-7)
Supplement: Supplementary file 3 — Additional file 3 Figure S2: Histological sections of adult female louse tissues from control samples and samples with selected genes knock-down. Figure S3: Histological sections of copepodids sampled three days post infection. [file 12864_2021_8054_MOESM3_ESM.pdf]

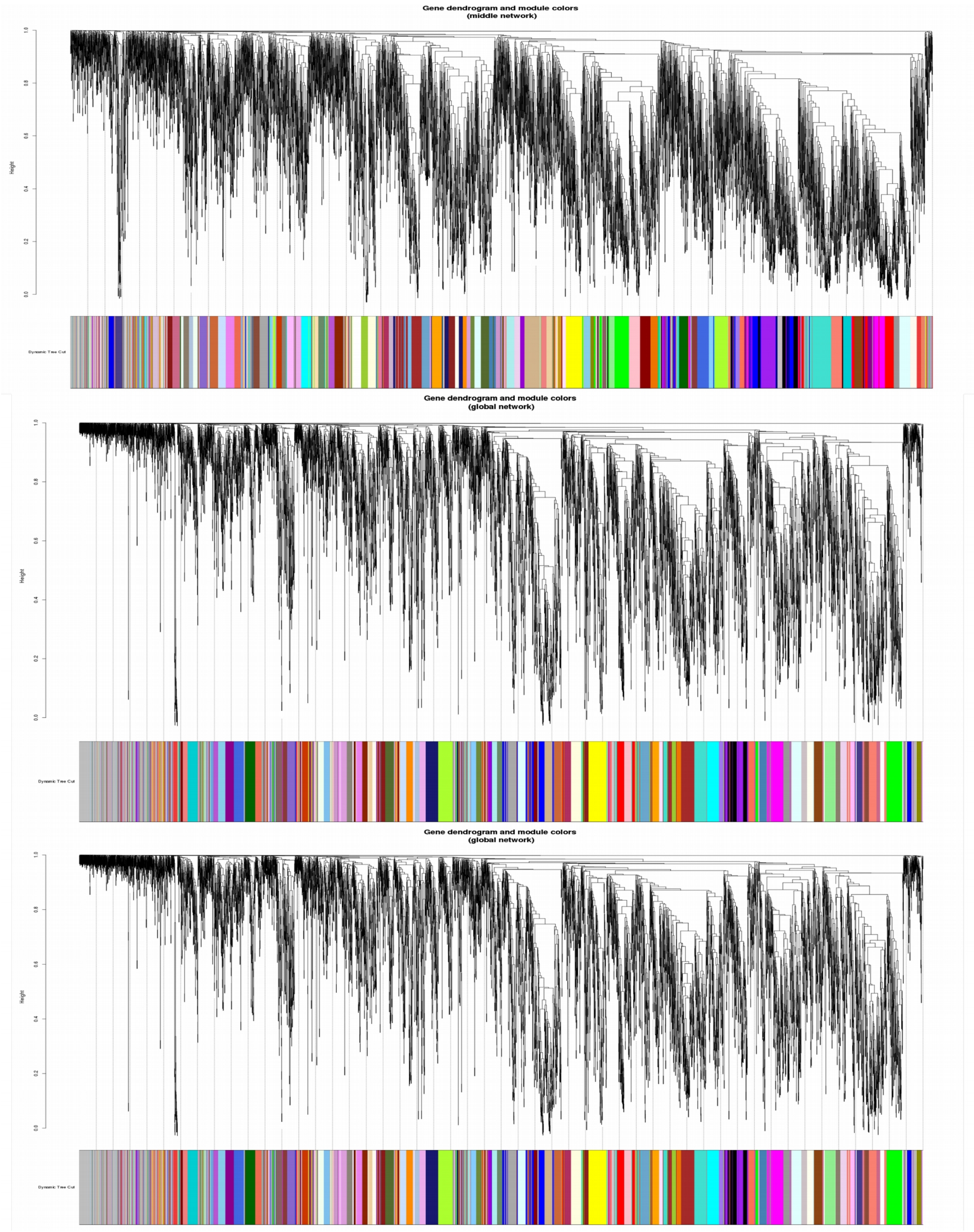

Figure S1: Hierarchical clustering tree (dendrogram) of nodes (transcripts) in the middle, moulting and global network. Each leaf (short vertical line) corresponds to one transcript. The color row below the dendrogram represents module membership of each transcript in the network.

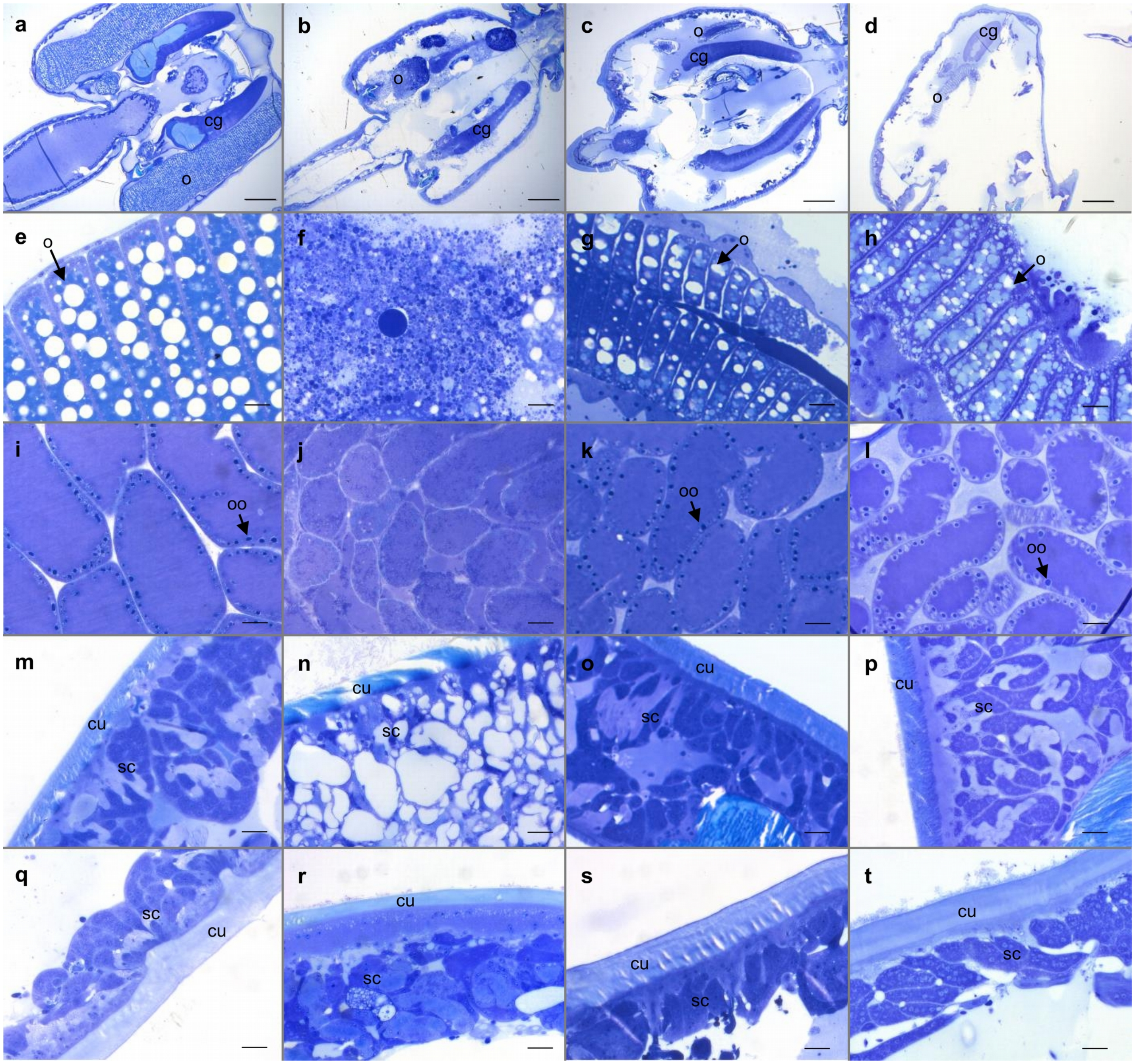

Figure S2: Histological sections of adult female louse tissues of a normal developed control louse with oocytes short before extrusion (first panel) and with unmatured oocytes (last panel), EMLSAG00000001458 knock-down louse (second panel) and EMLSAG00000004347 knock-down louse (third panel). Knock down was introduced in preadult-2 stage. Shown are the genital segment (a to d), oocytes (e to h), ovaries (i to l), the sub-cuticular tissue of the cephalothorax (m to p), and of the genital segment (q to t). o = oocytes, ce = cement gland, oo = oogonia; sc = subcuticular tissue, cu = cuticula; Scale bar: a-d = 500 µm, e-t = 10 µm.

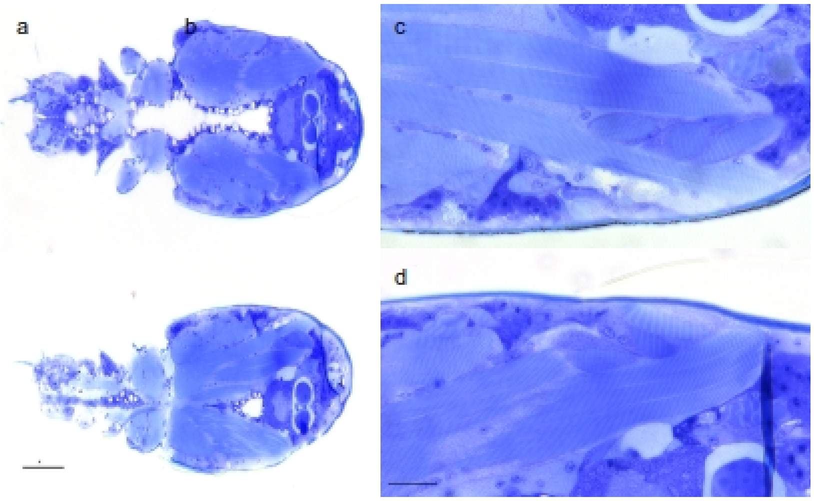

Figure S3: Histological sections of copepodids sampled three days post infection. Whole copepodids of control copepodid (a) and from EMLSAG00000001458 knock-down group (b). The scale bar corresponds 100 µm. Enlargements of muscle attachment place to cuticle are shown for control (c) and knock-down group (d). Scale bar corresponds to 50 µm.
